# Impact of pretransplantation malnutrition risk on the clinical outcome and graft survival of kidney transplant patients

**DOI:** 10.1590/2175-8239-JBN-2022-0150en

**Published:** 2023-07-10

**Authors:** Marina Ribeiro de Oliveira Santos, Marcus Faria Lasmar, Evaldo Nascimento, Raquel Aparecida Fabreti-Oliveira

**Affiliations:** 1 Hospital Universitário da Faculdade de Ciências Médicas, Belo Horizonte, MG, Brazil.; 2 Faculdade de Ciências Médicas, Belo Horizonte, MG, Brazil.; 3 IMUNOLAB – Laboratório de Histocompatibilidade, Belo Horizonte, MG, Brazil.

**Keywords:** Kidney Transplantation, Malnutrition, Nutrition Assessment, Renal Dialysis, Renal Insufficiency, Chronic, Transplante de Rim, Desnutrição, Avaliação Nutricional, Diálise Renal, Insuficiência Renal Crônica

## Abstract

**Background:**

The prevalence of malnourished patients before transplantation and the influence of malnutrition on graft and patient outcomes remain underestimated, despite being associated with higher postoperative morbidity and mortality. This study aimed to develop an easy nutritional screening tool and evaluate the impact of nutritional status on clinical outcome, graft survival (GS) and mortality risk in kidney transplant patients (KTP).

**Methods:**

In this retrospective cohort study including 451 KTP, we developed a score by using anthropometric, clinical, and laboratory measures performed in the pretransplant evaluation. The patients were stratified into 3 groups according to the final score: G1 (0 or 1 point)=low risk, G2 (2 to 4 points)=moderate risk, and G3 (>5 points)=high risk of malnutrition. The patients were monitored after transplantation at least 1 to 10 years.

**Results:**

Stratifying the 451 patients based on the pretransplant risk score, G1, G2, and G3 were composed of 90, 292, and 69 patients, respectively. Patients from G1 maintained the lowest serum creatinine levels at hospital discharge when compared with others (p = 0.012). The incidence of infection in the patients from G3 was higher than patients from G1 and G2 (p = 0.030). G3 recipients showed worse GS than G1 patients (p = 0.044). G3 patients showed almost threefold higher risk for graft loss (HR 2.94, 95% CI 1.084-7.996).

**Conclusions:**

KTP with higher malnutrition risk score were associated with worse outcomes and GS. The nutritional screening tool is easy to be used in clinical practice to evaluate the patient in preparation for kidney transplant.

## RESUMO

**Antecedentes:**

A prevalência de pacientes desnutridos antes do transplante e a influência da desnutrição nos desfechos do enxerto e do paciente permanecem subestimadas, embora estejam associadas a maior morbimortalidade pós-operatória. Este estudo buscou desenvolver uma ferramenta simples de triagem nutricional e avaliar o impacto do estado nutricional no desfecho clínico, sobrevida do enxerto (SE) e risco de mortalidade em pacientes transplantados renais (PTR).

**Métodos:**

Neste estudo de coorte retrospectivo incluindo 451 PTR, desenvolvemos um escore usando medidas antropométricas, clínicas e laboratoriais tomadas na avaliação pré-transplante. Os pacientes foram estratificados em 3 grupos segundo a pontuação final: G1 (0-1 ponto) = baixo risco, G2 (2-4 pontos) = risco moderado e G3 (>5 pontos) = alto risco de desnutrição. Eles foram monitorados por pelo menos 1 a 10 anos após o transplante.

**Resultados:**

Os 451 pacientes foram estratificados em G1, G2 e G3, que consistiram em 90, 292 e 69 pacientes, respectivamente. Os pacientes do G1 mantiveram os menores níveis de creatinina sérica na alta hospitalar em relação aos demais (p = 0,012). A incidência de infecção nos pacientes do G3 foi maior que nos pacientes do G1 e G2 (p = 0,030). Os pacientes do G3 apresentaram SE pior do que os pacientes do G1 (p = 0,044) e um risco quase três vezes maior de perda do enxerto (HR 2,94; IC 95% 1,084-7,996).

**Conclusões:**

PTR com maior escore de risco de desnutrição foram associados a piores desfechos e menor SE. A ferramenta de triagem nutricional é fácil de usar na prática clínica para avaliar pacientes em preparação para transplante renal.

## Introduction

Chronic kidney disease (CKD) is considered a worldwide public health problem with an increasing incidence and prevalence each year^1,2^. Annual costs for the treatment of CKD and end-stage renal disease (ESRD), including disease diagnosis and renal replacement therapy (RRT), and treatment of associated diseases are very high^3^. In patients with ESRD, malnutrition can occur in a large proportion, ranging from 18% to 75%^4^, as a consequence of several factors, and such patients usually present increased catabolism with reduction in lean body mass and fat^5,6,7,8^. In addition, a concomitant malnutrition-inflammation complex syndrome, an important risk factor for cardiovascular disease and mortality, can occur^9^. The nutritional status of these patients cannot be overlooked, being an important determinant of clinical outcomes in patients with CKD and one of the main predictor factors for morbidity and mortality in dialysis patients^10^. The best method for malnutrition diagnosis is still a matter of great discussion. Although the foregoing measures of nutritional status have practical value, each of these methods has limitations^11–13^.

In the last decades, graft and patient survival have improved; however, post-transplant complications remain high^5,14,15,16^. The demand for kidney transplants far exceeds the supply of available organs, causing a persistent increase in the number of patients on the waiting list with a parallel increase in the waiting time for cadaveric kidney transplant. Increasing long-term graft survival and reducing the need for a new transplant are paramount, not only in improving patient outcomes, but also for those awaiting a graft^17^. Patients on the waiting list or preparing for kidney transplantation often have significant nutritional changes and may become malnourished due to organ failure and associated symptoms. Following a successful kidney transplant, improved intake and gradual enhancement of adequate nutritional status are expected in these patients^18^.

The malnutrition in kidney transplant is associated with higher postoperative morbidity and mortality^19^. Some studies showed a prevalence of 15% to 23% of recipients with body mass index (BMI) less than 21^20^. In addition to post-treatment complications, such as rejections and infections, nutritional status may be an important determinant of clinical outcomes in transplant patients. Little is known about the role of malnutrition in kidney transplant recipients. Moreover, the prevalence of malnourished patients before and after transplantation and the influence of malnutrition on outcomes after the procedure are still underestimated. The aim of this investigation was to develop an easy-to-use nutritional screening tool based on scoring anthropometric, laboratory, and clinical data, and evaluate the impact of the nutritional status on the clinical outcome, graft survival and mortality risk in kidney transplant patients.

## Materials and Methods

### Patients and Study Design

This retrospective cohort study evaluated 451 kidney recipients (292 males and 159 females) with kidney from deceased or living donors. The recipients received a kidney transplant between 2008 and 2018 in the Transplantation Center of the University Hospital of the Faculty of Medical Sciences (UHFMS), Belo Horizonte, Minas Gerais, Brazil. The recipients aged >18 and <65 years who underwent clinical and laboratory evaluation and direct measurement of weight and height before the surgery. Patients with incomplete medical records and those involved in other clinical studies were excluded. This study was approved by the ethics committee of the Faculty of Medical Sciences (permit no. 2.122.409) and conducted based on principles of the Declaration of Istanbul. Informed consent has been obtained from the subjects and the procedures followed were in accordance with the Helsinki Declaration of 1975, as revised in 2013.

### Follow-up of Patients

The patients were monitored after transplantation at least 1 to 10 years as follows: weekly in the first month, every 15 days in the second month, every 30 days from the third month to the first year, every 2 months during the second year, every 3 months after the second year after transplant, and twice per year in the following years. At any time, additional ambulatory visits were made according to the patient needs. Serum creatinine levels were measured for graft function evaluation.

### Immunosuppression Therapy

The immunosuppression protocol used in the patients of this study was published by Lasmar et al.^21^. Briefly, induction immunosuppressive therapy with thymoglobulin (Genzyme, Mississauga, Canada) was used in retransplanted, hypersensitized, and sensitized patients with donor-specific anti-human leukocyte antigen antibodies (DSA). For maintenance therapy, a three-drug regimen that included tacrolimus (Libbs Laboratory, São Paulo, Brazil) or cyclosporine A (Biosintética, São Paulo, Brazil), corticosteroid prednisone (Eurofarm, São Paulo, Brazil), and mycophenolic sodium (Novartis, Basel, Switzerland) was used. In the presence of any adverse effect from calcineurin inhibitors detected by biopsy or in case of diarrhea, abdominal pain, weight loss, skin cancer, re-infection by cytomegalovirus, polyomavirus or papilloma virus, the change of medicationwas evaluate. These patients were converted to mycophenolic sodium and azathioprine (Laboratório Aspen Pharma, Serra/ES, Brazil). In patients with nephrotoxicity due to cyclosporine A or tacrolimus confirmed by renal biopsy, a switch was made to sirolimus (Laboratório Pfizer, São Paulo, Brazil) or everolimus (Laboratório Novartis, Basel, Switzerland). In those with important side effects such as proteinuria, lymphocele, and severe dyslipidemia linked to the use of sirolimus or everolimus, immunosuppression was converted to mycophenolic sodium or azathioprine. In patients with osteonecrosis, the drug prednisone was suspended^21^. The graft function was evaluated based on serum creatinine levels.

### Nutrition Score

We developed a practical score using pretransplant available data. The scores for pretransplant malnutrition risk (PMR) were calculated using anthropometric data, laboratory tests, and clinical conditions (Table 1).

**Table 1:** Pretransplant malnutrition risk score based on anthropometric, laboratory, and clinical data

**Anthropometric data**			
BMI (kg/m^2^)	BMI ≥ 22 □ 0 point	BMI 20–21.99 □ 1 point	BMI < 20 □ 2 points
**Laboratory data**			
Albumin	≥3.8 mg∕dL □ 0 point	3.4–3.79 mg∕dL □ 1 point	<3.4 mg∕dL □ 2 points
Serum cholesterol	≥120 mg∕dL □ 0 point	100–119.99 mg∕dL □ 1 point	<100 mg∕dL □ 2 points
Lymphocyte total count	≥1500 mg∕dL □ 0 point	800–1499 mg∕dL □ 1 point	< 800 □ 2 points
**Clinical data**			
Dialysis time (in years)	In dialysis for less than 1 year or preemptive transplant □ 0 point	In dialysis for over 1 year and less than 2 years □ 1 point	In dialysis for over 2 years □ 2 points
Comorbidities and dialysis time (in years)	No major comorbidities (not included in group I*) and non-diabetic □ 0 point	Diabetes mellitus with up to one target organ injury other than nephropathy □ 1 point	At least one comorbidity of group I * □ 2 points

Score 0-1 point: low risk (G1); 2-4 points: moderate risk (G2); score ≥ 5 points: high malnutrition risk (G3). *Comorbidities from group I: chronic obstructive pulmonary disease, coronary artery disease, heart failure, diabetes mellitus with more than 2 target organ lesions in addition to nephropathy, previous stroke.

Anthropometric data were assessed using BMI {ratio of dry weight in kilograms (kg)/height in meters squared (weight [kg]/height^2^ [m])}. The laboratory tests included serum albumin, cholesterol levels, and total lymphocyte count, which are biochemical markers suggestive of undernutrition and directly correlated with mortality in patients with CKD.

 These tests were performed by the UHFMS laboratory before the transplant procedure. Clinical data included preexisting comorbid conditions and time of patient on dialysis. The sum of all components of the PMR score ranged from 0 to 12 points. The patients were evaluated and stratified into three groups: group 1 (G1): 0 or 1 point, group 2 (G2): 2 to 4 points, and group 3 (G3): 5 or more points. A higher score showed a more severe pretransplant risk of malnutrition and inflammation.

### Statistical Analysis

Statistical analysis was performed using anthropometric, clinical, laboratory and immunogenetic information of recipients and their donors from databases with the SPSS analysis program for Windows version 18.0 (Chicago, IL, US). Differences were considered statistically significant if p value <0.05. The continuous numerical variables were submitted to normal distribution analysis by the Kolmogorov-Smirnov test. The means were compared using the F-test by analysis of variance. For variables with non-normal distribution, the comparison was made using the Kruskal-Wallis test. For the comparison of categorical variables, the chi-square test was used. Graft and patient survival analyses were performed using the Kaplan-Meyer method, and the comparison among the three groups was made by log-rank test. Cox multivariate model of proportional risks (hazard ratio – HR) was used to define predictive factors for the risk of graft failure. For the Cox regression analysis, the dependent variable was the time between the date of transplant to the last date of follow-up or occurrence of graft loss. The independent variables were demographic characteristics, clinical and laboratory data, and outcome. The significant independent variables (p>0.25) were used into the model by the hierarchical method. The HR (95% confidence interval) values were used to identify the effects of independent variables on the risk of graft loss. The importance of each variable in the model was assessed using the Wald test, and the assumption of proportionality of risk was assessed by analyzing the Schoenfeld residuals.

## Results

### Demographic Characteristics and Clinical Data

Based on these clustering criteria, the G1, G2, and G3 were composed of 90, 292, and 69 patients, respectively. The median time on RRT used as clinical data for grouping the patients was 9.0 (0 to 11), 20.5 (12 to 23), and 48.0 (24 to 73) months in G1, G2, and G3, respectively. The distribution of RRT type for G1, G2, and G3 respectively, was hemodialysis (75.56%, 94.48%, 89.86%), peritoneal dialysis (7.78%, 5.52%, 10.14%), and preemptive transplant (16.67%, 0.0%, 0.0%). The main causes of ESRD for patients from G1, G2, and G3 were, respectively, undetermined (47.78%, 46.05%, 49.28%), chronic glomerulonephritis (26.67%, 15.12%, 11.59%), diabetes mellitus (0.0%, 17.87%, 13.04%), autosomal polycystic kidney disease (11.11%, 6.19%, 7.25%), hypertensive nephropathy (5.56%, 9.62%, 11.59%), and others (8.89%, 5.15%, 7.25%).

We developed a score for the assessment of nutritional risk in pretransplant patients based on anthropometric, laboratory, and clinical data (Table 1). The demographic characteristics and clinical data of the patients are shown in Table 2. No statistical difference was found in the proportion of men and women in the three groups (Table 2). The mean age at the date of the transplant was 40.73, 44.85, and 45.71 for G1, G2, and G3, respectively. The patients from G2 and G3 had a mean age greater than those from G1 (p = 0.013) (Table 2). In G1, the majority of patients (84.4%) received a kidney from living donors, and in group 3, most of the patients (63.77%) received a kidney from deceased donors (p < 0.001) (Table 2).

**Table 2:** Demographic characteristics and clinical data of 451 kidney transplant patients according to score for nutritional status

Variable	G1	G2	G3	p value
**Number of patients**	90 (19.96%)	292 (64.74%)	69 (15.30%)	
**RECIPIENT**				
Sex				
Male	65 (72.22%)	179 (61.30%)	48 (69.57%)	0.109
Female	25 (27.78%)	113 (38.70%)	21 (30.43%)
***Receptor age (year) ± SD*** ***ABO blood group*** ***(n = 449)***	40.73 ± 12.432	44.85 ± 12.396	45.71 ± 12.884	**0.013**
O	43 (47.78%)	133 (45.86%)	37 (53.62%)	0.849
A	35 (38.89%)	111 (38.28%)	24 (34.78%)
B	7 (7.78%)	33 (11.38%)	5 (7.25%)
AB	5 (5.56%)	13 (4.48%)	3 (4.35%)
Retransplantation	2 (2.22%)	11 (3.77%)	6 (8.70%)	0.145
Risk of antibody-mediated rejection (n = 450)				
No sensitized	61 (68.54%)	174 (59.59%)	37 (53.62%)	0.379
Sensitized without DSA	23 (25.84%)	100 (34.25%)	26 (37.68%)
Sensitized with DSA	5 (5.62%)	18 (6.16%)	6 (8.70%)
Mean % PRA Class I	8.00 ± 20.78	9.93 ± 21.82	10.90 ± 22.83	0.678
Mean % PRA Class II	4.95 ± 15.13	6.43 ± 19.13	7.13 ± 18.85	0.732
**DONOR**				
Donor age (year) ± SD	39.67 ± 10.949	43.29 ± 12.649	42.52 ± 13.734	0.056
Donor type				
Living	76 (84.44%)	134 (45.89%)	25 (36.23%)	**<0.001**
Deceased	14 (15.56%)	158 (54.11%)	44 (63.77%)
**TRANSPLANT PROCEDURE**				
For deceased donor (n = 216)				
Cold ischemia time (h) ± SD	14.185 ± 8.1407	16.989 ± 6.5732	16.565 ± 5.9264	0.332
Expanded criteria	5 (35.71%)	36 (22.78%)	12 (27.27%)	0.520
HLA-A, -B, -DRB1 mismatching (n = 449)				
0	19 (21.35%)	21 (7.22%)	5 (7.25%)	**0.003**
1 to 3	43 (48.31%)	161 (55.33%)	40 (57.97%)
4 to 6	27 (30.34%)	109 (37.46%)	24 (34.78%)
rATG immunotherapy induction	12 (13,33%)	56 (19,18%)	17 (24,64%)	0.190

G: group; SD: standard deviation; DSA: donor-specific antibody. p values <0.05 are indicated in bold.

No statistical difference was found among the three groups for the variables donor age, ABO blood group, retransplantation, and risk for antibody mediated-rejection (Table 2). For patients who received a kidney from a deceased donor, no statistical differences were found among the three groups for cold ischemia time and transplantation with donor with expanded criteria (Table 2). Considering the HLA-A, -B, and -DRB1 compatibility, based on the number of HLA mismatches (0 to 6), patients from G1 had better HLA compatibility with their donors than patients from G2 and G3 (p = 0.003) (Table 2).

### Outcomes Associated with Nutrition Score

The median follow-up time was 48 months, with a minimum and maximum of 2 and 120 months, respectively. No statistical difference was observed in patients with delayed graft function (DGF) incidence for those recipients that received a kidney from a deceased donor (Table 3). The proportion of infection episodes by cytomegalovirus, urinary tract infection by any etiologic agent, and polyomavirus was not statistically different among the three groups (Table 3). However, when the incidence of infection and the immunotherapy induction were analyzed at the same time, patients from G3 had a higher proportion of infections (35.1%) compared to patients from G1 (14.6%) and G2 (20.3%) (p = 0.030). The rejection proportions in the first year were not statistically different among the groups, despite the trend toward higher proportions observed in G2 and G3 than in G1 (Table 3). However, patients in G3 lost their grafts more than those in G2 and G1, mainly due to immune cause or infection (p = 0.038) (Table 3).

**Table 3:** Outcomes in transplanted patients with different nutritional profiles before transplantation

Variable	G1 (n = 90)	G2 (n = 292)	G3 (n = 69)	p value
**DGF for deceased donor (n = 216)**	7 (50.00%)	94 (59.49%)	31 (70.45%)	0.284
**Main infections**	48 (53.33%)	182 (62.33%)	37 (53.62%)	0.188
*Cytomegalovirus*	10 (20.83%)	37 (20.33%)	6 (16.22%)	NA
*UTI*	28 (58.33%)	89 (48.90%)	16 (43.24%)
*Polyomavirus*	0 (0%)	6 (3.30%)	2 (5.41%)
**Rejection episodes in the first year**	19 (21,11%)	76 (26,03%)	19 (27,54%)	0.577
*TCMR*	13 (68.42%)	65 (85.53%)	14 (73.68%)	NA
*AMR*	6 (31.58%)	8 (10.53%)	5 (26.32%)
*TCMR + AMR*	0 (0.00%)	3 (3.95%)	0 (0.00%)
**Graft loss caused by**	17 (18,89%)	77 (26,37%)	23 (33,33%)	**0.038**
Immune cause*	1 (5.88%)	17 (22.08%)	8 (34.78%)	NA
*Infection*	5 (29.41%)	20 (25.97%)	9 (39.13%)
Other**	10 (58.82%)	35 (45.45%)	5 (21.74%)
*Missing data*	1 (5.88%)	5 (6.49%)	1 (4.35%)

DGF: delayed graft function; UTI: urinary tract infection; NA: not analyzed; TCMR: T cell-mediated rejection; AMR: antibody-mediated rejection; NA: not analyzed. *Immune cause: TCMR, AMR, and IFTA (interstitial fibrosis and tubular atrophy). **Other: delayed graft function, vascular thrombosis, and cardiovascular disease. p values <0.05 is indicated in bold.

**Figure 1.**
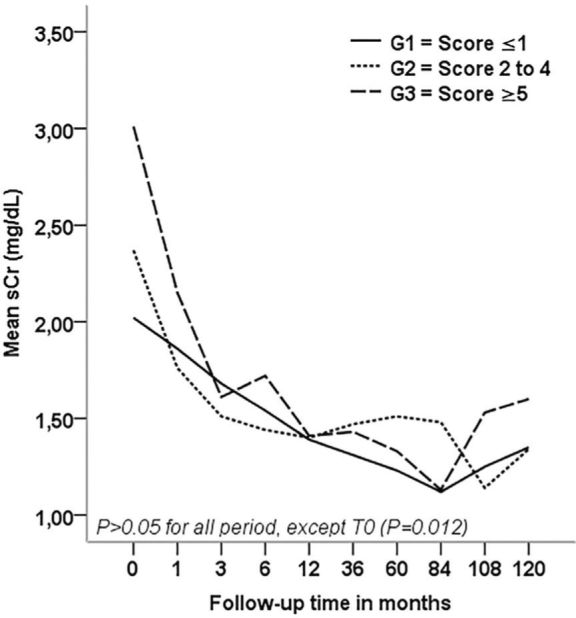
Renal function for patients who did not lose their graft (T = 0 is hospital discharge).

**Figure 2.**
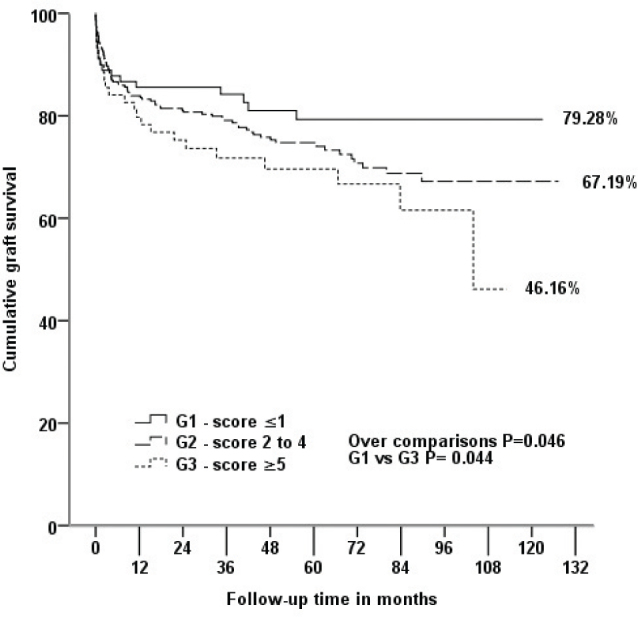
Kaplan-Meier survival curve for the three groups analyzed over 10 years of follow-up.

**Figure 3.**
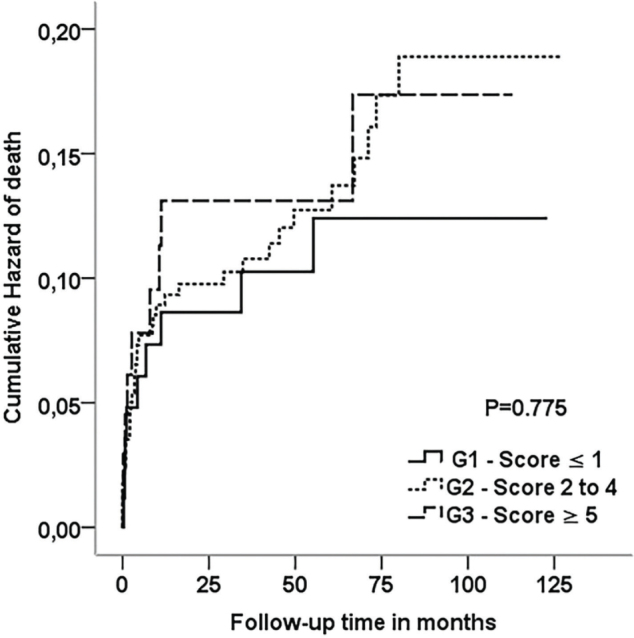
Mortality risk in the three groups analyzed over 10 years.

In recipients who did not lose their graft, patients from G1 were able to maintain lower serum creatinine levels when compared with patients from G2 and G3 at hospital discharge (p = 0.012). More similar kidney functions were observed mainly in the first year after transplantation in patients of the three groups (Figure 1). Kaplan-Meier survival curves (Figure 2) showed that graft survival was statistically different among the groups in over comparison analysis (p = 0.046). Patients from G1 had better graft survival than those from G3 (p = 0.044). The estimated means in months for graft survival time were 100.56 ± 46.49, 94.64 ± 54.34, and 77.76 ± 49.01 for G1, G2, and G3, respectively. Although the differences in mortality risk over ten years were not statistically significant, a trend of a lower mortality risk in G1 patients than in G2 and G3 patients was observed (p = 0.775) (Figure 3).

In univariate Cox regression analysis of the association between graft loss and covariates, a significant relationship was found with donor age, retransplant, patients from G3, sensitized patients without DSA who did not receive rATG immunotherapy, those who received kidney from deceased donor, patients with DGF, patients who received an immunosuppressive drug other than TAC or CSA therapy, and those who had T cell-mediated rejection (TCMR) or antibody-mediated rejection (AMR) (Table 4). Of these, the following significant predictors remained on multivariate analysis: patients from G3 with high malnutrition risk, sensitized patients without DSA, those who have had DGF, and patients who have had TCMR or AMR rejection episodes (Table 4). With regard to the risk for graft loss, G3 showed almost threefold higher risk (hazard ratio [HR] 2.94; 95% confidence interval [CI] 1.084-7.996), and sensitized patients without DSA who did not receive rATG immunotherapy and patients with DGF had almost twofold higher risk (HR 1.904, 95% CI 1.168-3.105; HR 1.921, 95% CI 1.238-2.980). Patients with TCMR or AMR rejection had a 2.18-fold higher risk (HR 2.180, 95% CI 1.251-3.798) (Table 4).

## Discussion

In this retrospective cohort study, 451 kidney transplant recipients were followed-up in median of 48 months. We developed a PMR score for these patients and found that almost 80% of the kidney recipients were classified as moderate to high risk of malnutrition. Malnutrition is highly prevalent in ESRD patients on hemodialysis treatment, and it is associated with hospitalization and death^22^. However, data regarding the actual prevalence and incidence in transplant patients, especially during the first post-transplant year, and their relationship with graft and patient outcomes are underestimated. The immediate post-transplant period is considered the critical phase because the patient is recovering from the surgical procedure and taking high doses of immunosuppressant medications. The body needs to treat protein catabolism, promote wound healing, and treat electrolyte abnormalities. Malnutrition at this time is associated with impaired surgical wound healing and higher risk of infection^23,24^.

About 85% of the patients from G1 received a kidney from living donors compared with 46% and 36% of the patients from G2 and G3, respectively. Thus, patients from G1 had less time on hemodialysis and were transplanted younger than patients from G2 and G3, thereby reducing the risk of becoming malnourished. In addition, patients from G1 transplanted with a living donor had better HLA compatibility with their donors than patients from G2 and G3. Immunotherapy induction using rATG in malnourished patients from group 3 increased the incidence of post-transplant infections by cytomegalovirus, urinary tract infection, and polyomavirus. It is important to highlight that the percentage of patients with diabetes in each group seems to be lower than expected and this may be due to the fact that many diabetic patients do not have an early diagnosis being often classified as CKD of uncertain etiology.

Evaluation of nutritional risk, one of the strongest predictors of morbidity and mortality in CKD patients, is a difficult and frequently forgotten process^25^. Kalantar-Zadeh et al. developed the malnutrition scoring system (MIS) for evaluation of the severity of malnutrition-inflammation complex syndrome on maintenance dialysis therapy^26^. This system was already used to evaluate malnutrition in different stages of chronic disease and showed an association with mortality in patients with CKD. It is also considered a significant predictor of mortality in kidney transplant patients^27,28^. MIS is recommended to use a combination of clinical measures, as well as laboratory tests to assess nutritional status. Serum albumin, serum cholesterol level, and total lymphocyte counts are considered markers for nutrition status, and their low levels are associated with increased risk of mortality in patients with ESRD^6,29,30^. Hypoalbuminemia has been linked to poor clinical outcomes in all stages of CKD with higher hospitalization indices and mortality. Therefore, serum albumin can be a useful marker of nutritional and clinical status^12,27,31^. Anthropometry may be used as a helpful tool when malnutrition is suspected in patients with CKD. Among the anthropometric measures, BMI is the most commonly used, and it is also a predictor for increased risk of mortality in patients undergoing regular dialysis^26,32^. Extreme BMI values can be related to higher mortality of kidney recipients^33^. The cholesterol serum can be used as a caloric depletion parameter and previous reports have shown an association between low cholesterol serum and mortality in dialysis patients^34,35^. Total lymphocyte count is used as an indicator of the loss of immune defenses caused by undernutrition and it has been used as a useful marker in other nutritional scoring tools^36^. There is no single marker capable of predicting the risk of malnutrition. Thus, what is recommended is the association of several parameters in the search for a more accurate diagnosis^10,37,38^. Over the past years, we have been able to accompany the creation and validation of some scoring systems for the nutritional classification of these patients, but the regular assessment of complete clinical parameters is time consuming and not practical in the routine pretransplant evaluation. Therefore, the use of a simple nutrition screening can be very helpful. Developing an easy, simple, and low cost nutrition screening tool is clinically valuable for pretransplant kidney patients to identify their nutritional risk. A tool that can also be used by all nephrologists, based on routine objective measurements, such as anthropometric, laboratory, and clinical data, is important. In our experience, this study appears to be the first to evaluate the predictive power of poor nutritional status on graft and patient outcomes by using a simple score based on routine objective measurements.

The incidence of DGF observed in our study was higher, mainly in patients with higher malnutrition risk. DGF is associated with several complications in post-transplant patient care and with poor allograft survival. Molnar et al. studied 8961 patients and showed that lower levels of albumin before kidney transplantation are associated with worse short- and long-term post-transplant outcomes, including higher risk of DGF and mortality^39^.

Patients with higher risk of malnutrition in this study were associated with lower allograft survival rate and higher incidence of infections when the patient was induced with rATG. Our findings are consistent with previous studies^40,41,42,43,44,45^. Hwang et al. studied kidney transplant recipients using a different pretransplant score (malnutrition, inflammation, and atherosclerosis score – MIA) and found an association with higher MIA score with lower albumin levels and the occurrence of post-transplant acute coronary syndrome^43^. Improving allograft function is essential to decrease the risk of graft failure, reducing the need for retransplantation, and to improve patient’s survival. This study has several strengths, including its design and the relatively notable size of kidney transplant patients with 10 years of follow up. Some limitations of this study were the retrospective nature, single-center cohort and observational study. In addition, no dietary intervention was made. However, despite the limitations, this study has the potential to be of great importance and application for the pretransplant evaluation of recipients.

In conclusion, patients with higher malnutrition risk scores were associated with worse outcomes and poor allograft survival. This study highlights the importance of nutrition screening to identify malnutrition as early as possible in pretransplant patients. Predicting short-term outcomes in kidney transplantation can be useful to foresee long-term results and reduce the need for retransplantation. Future studies are necessary to better elucidate the metabolic changes and special nutrient demands in this period and to further explore the benefits of nutrition intervention on pre- and post-transplant outcomes.
