# Erratum: Impact of pretransplantation malnutrition risk on the clinical outcome and graft survival of kidney transplant patients

**DOI:** 10.1590/2175-8239-JBN-2022-0150eren

**Published:** 2023-12-22

In the article “**Impact of pretransplantation malnutrition risk on the clinical outcome and graft survival of kidney transplant patients**”, with DOI code number https://doi.org/10.1590/2175-8239-JBN-2022-0150en, published in the Brazilian Journal of Nephrology (Jornal Brasileiro de Nefrologia) *ahead of print*, 2023, it was missing Table 4:

**Table 4:** Predictive factors associated with the occurrence of graft loss

Variable	Univariate analysis				Multivariate analysis			
	HR	95% CI for HR		p value	HR	95% CI for HR		p value
Receptor age	1.006	0.991	1.021	0.418				
Donor age	1.022	1.006	1.037	0.005	–	–	–	–
Male	1.005	0.689	1.466	0.979				
Retransplantation	1.555	0.757	3.193	0.229	–	–	–	–
HLA-A,-B,-DRB1 MM								
*0*	Reference							
*1 to 3*	1.388	0.692	2.786	0.356				
*4 to 6*	1.387	0.675	2.849	0.374				
Pretransplant malnutrition risk score								
*G1 – Score 0–1*	Reference							
*G2 – Score 2–4*	1.43	0.845	2.418	0.183	1.506	0.613	3.696	0.372
*G3 – Score ≥5*	1.881	1.005	3.522	0.048	2.944	1.084	7.996	**0.034**
Risk of AMR								
*Non sensitized*	Reference							
*Sensitized without DSA*	1.343	0.917	1.967	0.13	1.904	1.168	3.105	**0.010**
*Sensitized with DSA*	1.38	0.685	2.779	0.368	1.045	0.434	2.520	0.921
Deceased donor (vs living donor)	2.081	1.43	3.028	0.051	–	–	–	–
Expanded criteria	1.309	0.782	2.191	0.305				
Cold ischemia time	1.008	0.971	1.047	0.678				
Delayed graft function	2.583	1.789	3.729	<0.001	1.921	1.238	2.980	**0.004**
Immunosuppression								
*TAC+MYF+CP*	Reference							
*CSA+MYF+CP*	0.83	0.455	1.511	0.542	–	–	–	–
*Other*	1.654	0.722	3.786	0.234	–	–	–	–
Induction therapy	1.047	0.658	1.664	0.847				
TCMR or AMR rejection	2.109	1.467	3.033	<0.001	2.180	1.251	3.798	**0.006**
Infection episode	0.991	0.684	1.437	0.963				

HR: hazard ratio; MM: mismatch; TAC: tacrolimus; CSA: cyclosporine A; CP: corticosteroid prednisone; TCMR: T cell-mediated rejection; AMR: antibody-mediated rejection.

Variables with p ≤ 0.25 in univariate analysis were used to construct the Cox multivariate analysis. p values <0.05 are indicated in bold.

